# A Heterogeneous Layer-Based Trustworthiness Model for Long Backhaul NVIS Challenging Networks and an IoT Telemetry Service for Antarctica

**DOI:** 10.3390/s21103446

**Published:** 2021-05-15

**Authors:** Adrià Mallorquí, Agustín Zaballos

**Affiliations:** GRITS, Engineering Department, La Salle, Universitat Ramon Llull (URL), 08022 Barcelona, Spain; agustin.zaballos@salle.url.edu

**Keywords:** trustworthiness, model, telemetry, IoT, NVIS, challenging network, Antarctica

## Abstract

Antarctica is a key location for many research fields. The lack of telecommunication systems that interconnect remote base camps hardens the possibility of building synergies among different polar research studies. This paper defines a network architecture to deploy a group of interconnected remote Antarctic wireless sensor networks providing an IoT telemetry service. Long backhaul NVIS links were used to interconnect remote networks. This architecture presents some properties from challenging networks that require evaluating the viability of the solution. A heterogeneous layer-based model to measure and improve the trustworthiness of the service was defined and presented. The model was validated and the trustworthiness of the system was measured using the Riverbed Model simulator.

## 1. Introduction

During the last half-century, Antarctica has been a key location for many research studies in several fields such as oceanography, bioscience, geoscience, physical sciences, and other environmental studies [1]. Although many bases have been settled in the peripheral areas of the Antarctic continent [2], the difficult environment and terrain provoke numerous challenges when it comes to implementing new operational services for modern studies. One of these challenges is the lack of telecommunication systems in Antarctica [3], especially wireless sensor networks (WSNs). Without WSNs, new research studies tend to use non-automatized ways of gathering data, which are more complex logistically, less scalable, and more error prone. Moreover, most Antarctic bases are not interconnected [3]. This fact lowers the possibilities for different research groups to collaborate on similar studies, and the advantages of providing synoptic region-wide observations and building synergies are lost [3].

The lack of conventional telecommunication services in Antarctica leverages the use of satellite communications or other systems such as high-frequency (HF) links to build a network of interconnected remote WSNs [4]. The first option is commonly discarded because of economic reasons, given the high costs of subscribing to this type of service. Furthermore, the degree of coverage offered by satellite constellations in Antarctic latitudes is not desirable [4]. To overcome these difficulties, the SHETLAND-NET [5] project aims to expand the use of communications in HF (3–30 MHz) by ionospheric reflection to the establishment of a low consumption communications system that allows the collection of sensor data distributed throughout the archipelago of the South Shetland Islands. This technology, called near vertical incidence skywave (NVIS), does not require direct vision and is totally independent of the satellite since the signal is transmitted upwards, allowing it to overcome any geographical feature [4,6,7]. The long backhaul NVIS link has a coverage range of up to 250 km, and its reliability is dependent on ionospheric conditions and solar activity. Researchers from our university have previously participated in research campaigns on Livingston Island, studying and verifying the NVIS communication system’s viability [8,9,10]. A new campaign is planned to be carried out in the Antarctic field, with the goal to test the new improvements of the NVIS link [11] and deploy an IoT network for three different use cases: a telemetry service for light data (e.g., penguin tracking [12], a telemetry service for fat data (e.g., lichen observation [13]), and a distributed computing service to map the ionosphere along Antarctica).

However, a network deployed with long backhaul NVIS links may present some situations typical of challenging networks [14], such as intermittent connectivity, end-to-end disconnection, and variable error rates, making the implementation of the aforementioned services over a traditional TCP/IP architecture difficult. For the sake of the project, it is not feasible to wait until the Antarctic campaign starts to test the system in the field. Antarctic campaigns are usually very time restricted due to the meteorological conditions. Its remote location makes it challenging to overcome unanticipated difficulties that may arise (e.g., incorrect dimensioning of the needed equipment, poor performance of the proposed architecture). For this reason, it is necessary to study the viability and the expected trustworthiness of implementing this kind of network before its deployment in the field. The pre-deployment phase of the SHETLAND-NET project needs this previous research on the factors that affect the robustness of a communication network, which will help us build more reliable expectations for our proposed service’s results and minimize the number of unexpected adversities (e.g., degraded service performance and reliability, loss of connectivity). In our case, this study was executed by simulating the conditions and the service that will be deployed in Antarctica.

This paper focuses on the use case of the telemetry service for light data. Many Antarctic studies could be helped by automating the data gathering of their research (e.g., geomagnetic studies [15], blowing snow monitoring [16], climate change [17], biological monitoring [12], or permafrost analysis [18]). Most of the data for these studies are currently gathered manually, and some zones might be challenging to reach, even with special vehicles such as snow motorbikes. For these reasons, the studies are focused on small areas of the Antarctic region. Thus, a WSN that provides a broader coverage area and the interconnection of remote areas could increase the results’ relevance (e.g., more samples could be collected, broader synergies could be built). Moreover, the long backhaul links in charge of communicating remote WSNs could also be used to interconnect different Antarctic bases [4].

The paper has two main objectives. First, it was necessary to define which architecture, technologies, and protocols the telemetry service will use. As mentioned before, the drawbacks of challenging networks in addition to the extreme conditions sensors and other equipment need to work within is that it can provoke the service to reach low levels of performance and trustworthiness in the face of adversities. Thus, the paper’s second objective was to propose and validate a model for visualizing, understanding, and measuring the trustworthiness of the overall service before its deployment in the field. With this model, the service’s weaknesses could be detected, and countermeasures could be proposed to improve its trustworthiness and foresee their impact. We used the Riverbed Modeler simulator [19] to validate the model and measure the service’s trustworthiness. To confirm the results, the tests were performed by modeling the permafrost use case of [18], where Ground Terrestrial Network-Permafrost (GTN-P) stations were used to measure 32 different parameters. These tests can be replicated to other concrete telemetry use cases by modeling them too.

The rest of the paper is organized as follows. In Section 2 and Section 3, the background and related work are described, respectively. Section 4 defines the use case’s service architecture. Section 5 presents the trustworthiness model. In Section 6, the performed simulations are described, and the extracted results are discussed in Section 7. Finally, the conclusions of the paper and future work are detailed in Section 8.

## 2. Background Work in IoT

This section presents mature IoT and WSN technologies that can help to define the network architecture of our telemetry use case for remote regions. In terms of network architecture for WSNs, it is necessary to differentiate between the access network and the backbone network. On the one hand, the access network provides connectivity to the IoT sensors in a variable coverage range, depending on the technology. On the other hand, the backbone is in charge of interconnecting the access networks to build a global WSN. The backbone network can use long backhaul links to reach remote areas and broader coverage than access network technologies.

### 2.1. IoT Access Network

The access network technologies for WSNs are commonly known as the IoT communication protocols [20] or IoT MAC layer protocols [21]. These protocols are commonly classified, depending on the size of the coverage area, as short-range coverage protocols and long-range protocols. Networks built on the latter kind of protocols are commonly known as low-power wide area networks (LPWANs). The authors of [20,21] classified the most used technologies in IoT. For short-range networks, the most common technologies are RFID, NFC, Bluetooth Low Energy (BLE), Zigbee, 6LoWPAN, and Z-Wave. For LPWANs, the most used communication protocols are narrow-band IoT (NB-IoT), long-term evolution-enhanced machine-type communication (LTE eMTC), Sigfox, and LoRa. To the best of our knowledge, in the specific case of Antarctica, the deployment of WSNs are scarce and limited to temporary testing deployments but not persistent. One example of short-range communications is the SNOWWEB project [22], where a network of weather stations was built using Zigbee transceivers. LPWANs seem to be more suitable options since the coverage area for deploying the WSN is more extended. For that reason, the authors of [23] studied the applicability of LoRa in Antarctic regions by characterizing its channel in the field, achieving a coverage area of up to a 30 km radius. Despite this, it seems feasible that some sensors of the WSN can be located out of range of the gateway due to the geographic conditions. In this case, there is the need to use mobile gateways and deploy mobile ad hoc networks (MANETs) [4,24,25,26].

### 2.2. IoT Backbone Network

On the other hand, the backbone network is in charge of interconnecting remote WSNs to build a single major network. For this purpose, LPWAN communications are not valid because the links that need to be established must have a broader range (several tenths of kilometers). Moreover, since the Antarctic region has many terrain variations, it is expected that two nodes separated by several kilometers do not have line of sight (LOS) [7]. Satellite communications are a solution to overcome these problems. However, geostationary Earth orbit (GEO) satellites do not cover Antarctica’s latitudes adequately, and current low-altitude Earth orbit (LEO) satellites provide partial or no coverage in deep polar regions [27]. The authors of [27] studied the possibility of covering the whole Antarctic continent with a three-satellite constellation in elliptical orbits, but it has not been implemented. A significantly lower cost solution suitable for WSNs in remote areas is the use of HF communications. Specifically, the NVIS technique has already been tested in Antarctica [4,6,7]. Results show that this kind of long backhaul link can reach a throughput of up to 20 Kbps and a coverage radius of up to 250 Km without the need for LoS [28]. The main drawback of NVIS is the considerable variation of the transmitting channel’s characteristics, the ionosphere, which can lead to periods of non-connectivity, becoming a challenging network [14]. Thus, it is necessary to test and measure NVIS networks’ trustworthiness when used to transport data from actual use cases.

## 3. Related Work on Cyber Physical Systems’ Trustworthiness

This section describes the related work by other authors to define and measure the trustworthiness of cyber physical systems (CPS). A CPS is defined as a system with integrated computational and physical capabilities. Common examples of CPSs include industrial control systems, automated vehicles and aircraft controls, wireless sensor networks, smart grids, and almost all devices typically encompassed by the Internet of Things [29,30]. The trustworthiness of CPS is defined in the literature, in general terms, as the property of behaving as expected under adversarial conditions [31]. However, these adversarial conditions can come from different reasons, e.g., faulty nodes, byzantine errors, malicious behaviors, and network malfunction [32]. For this reason, in the literature, there can be found many different approaches to measuring or providing trustworthiness that refer to disparate elements. We propose to classify them into the following four categories that will be used later to define our trustworthiness model:

1. Data Trustworthiness: It is defined as the possibility of ascertaining the correctness of the data provided by the source [33]. Many methods try to detect faulty nodes, false alarms, and sensor misreading using different approaches [32]. For instance, the authors of [34] presented a distributed Bayesian algorithm to detect faulty nodes, while the authors of [35] used a fog computing architecture to detect, filter, and correct abnormal sensed data. In addition, the authors of [36] presented a data intrusion detection system to trigger false data from malicious attacks;
2. Network Trustworthiness: It can be defined as the probability that a packet will reach its destination unaltered despite the adversities (e.g., link failure, link saturation, malicious attacks), and it is a crucial factor of low-power and lossy networks (LLNs) [37]. Improving network trustworthiness and performance is a challenge that has been addressed from different perspectives such as transmission coding [38,39,40,41], load balancing and redundancy protocols [42], transport protocols [43], dynamic routing and topology control protocols [44,45], cybersecurity mechanisms [46], and delay tolerant network (DTN) architectures and protocols [47]. In the case of routing, both proactive routing protocols (e.g., the IPv6 Routing Protocol for low-power and lossy networks (RPL) and optimized link state routing (OLSR)) and reactive routing protocols (e.g., ad hoc on-demand distance vector (AODV) and link-quality source routing (LQSR)) have been proposed in the literature to solve the drawbacks of LLNs and MANETs [44,45];
3. Social Trustworthiness: This trend has gained more attention since the irruption of the Social Internet of Things (SIoT) concept [48,49]. In SIoT trustworthiness, the capability of the objects to establish social relationships autonomously between them is leveraged to define more complex trust and reputation models that take into account several input parameters. The authors of [50] define a subjective model that considers factors as the computational capabilities of the nodes, the type of relationship between them, the total number of transactions, the credibility of a node, and the feedback provided by other nodes, among others. In [51], the authors evolved their previous model and based it on more parameters, such as the neighborhood of nodes, and presented a new objective model with a faster transitory response. The authors of [52] proposed another model that defines the input parameters as the expected gain on success, the expected damage on a failure, the expected cost, the expected result, and the goal. The authors of [53] define a decentralized, self-enforcing trust management system based on a feedback system and reputationally secure multiparty calculations to ensure the privacy of each party’s provided data;
4. Consensus: This represents a state where all participants of the same distributed system agree on the same data values [54]. Consensus protocols can be divided into two general blocks: proof-based consensus and byzantine consensus. The first group is oriented to blockchain technology, where all participants compete with each other to mine a block, and the most used protocols are proof-of-work, proof-of-stake, and its variants [55,56,57,58,59]. The main drawback of these protocols for IoT is that most devices have simple hardware specifications and low processing power, being incapable of performing the mining tasks of blockchain [60]. The second major group of consensus protocols is the more classical byzantine based. These kinds of protocols implement voting-based mechanisms to reach an agreement rather than competing among them, which generally results in less resource consumption. The drawback of these mechanisms is the number of messages that need to be delivered through the network to reach an agreement. The most well-known protocols in this category are Practical Byzantine Fault Tolerance, RAFT, PaXoS, and Ripple, although several variants have emerged year-by-year [55].

To the best of our knowledge, all the approaches that can be found in the literature focus on specific areas of trustworthiness, but none of them include all of the four trustworthiness topics. This fact can lead to misinterpreting the reasons for an inferior service’s trustworthiness level, and wrong countermeasures to improve it could be applied if the interdependencies between different trustworthiness categories are not considered (as will be seen in Section 5.4). For this reason, we found the need to design our own model to measure a system’s trustworthiness level that included the four categories mentioned above, which could help us anticipate and identify the possible weaknesses in our IoT telemetry system. Table 1 summarizes the characteristics of the analyzed trustworthiness approaches.

## 4. Network and Service Architecture

Prior to applying the model of trustworthiness, our first goal was to define the architecture of the telemetry use case that was to be deployed in the Antarctic campaign of the SHETLAND-NET project. As mentioned before, the concrete case was the improvement of permafrost studies by automating data gathering from the GTN-P stations (the sensors), which measure 32 different parameters. Currently, data are gathered only once a day, and the authors from [18] left the complete automation of the GTN-P stations as an open challenge, given that their approach suffers from a lack of connectivity. It is important to remark that the architecture described below applies to any telemetry use case. However, we will use the example of the GTN-P stations’ permafrost research that will be carried out during our campaign in the field.

**Figure 1:**
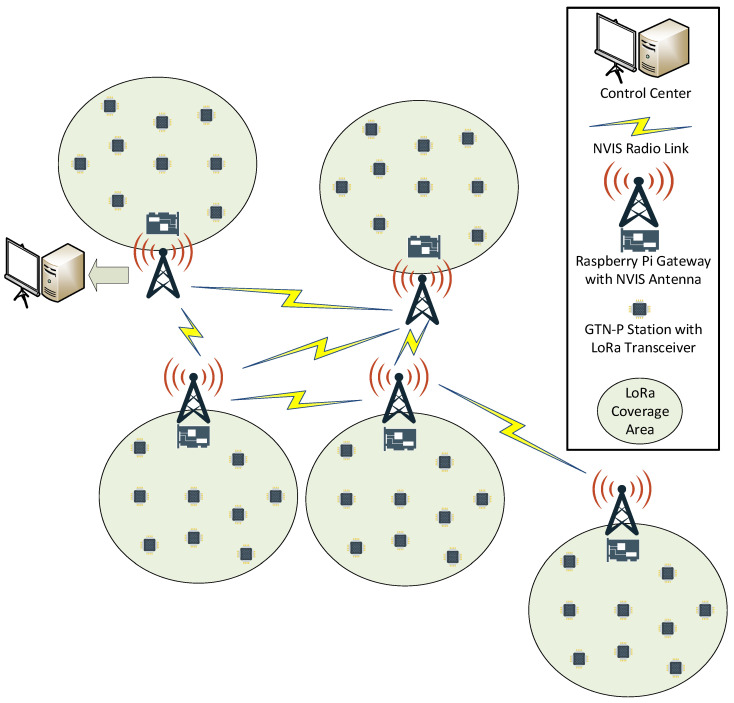
Network diagram of the SHETLAND-NET project telemetry service.

We propose to use the architecture defined for the deployment phase SHETLAND-NET project [5]. In our approach [28], we aim to interconnect all remote sensors to a control center, building a heterogeneous global wireless sensor network (GWSN) composed of several wide wireless sensor networks (WWSN), able to gather data more frequently. The first approach to designing a remote sensing system for the Antarctic region was described in [4] during the SHETLAND-NET project’s early stages, describing how sensors could reach and use NVIS as a long backhaul link. However, it was mostly centered on designing the characteristics of the physical layer of the NVIS (backbone) network. In this paper, a more detailed description of the overall network architecture is presented. The network diagram is detailed in Figure 1.

The access network (WWSN) will be in charge of providing connectivity to the remote sensors, transporting the gathered data from the sensors (GTN-P stations connected to a low-consumption board) to the gateways (e.g., a Raspberry Pi). The main gateway of each WWSN will be located near the research base, with the GTN-P stations located around it in a few-kilometer radius. For redundancy reasons, groups of GTN-P stations can be clustered and placed close enough to interpret that they measure the same permafrost values. These stations will sense the data and send it to the gateway once per hour. For this use case in Antarctica, it is logical to think that the wider the area that can be covered by the access communication technology the better, because more sensors will be able to be placed far from the research base so researchers will have access to sensors placed farther away while saving valuable time in collecting the data. For this reason, short-range communications are less suitable, and LPWAN communications are preferred. The lack of telecommunication operators providing service in Antarctica forces operator-dependent communication services, such as Sigfox, NB-IoT, or LTE eMTC, to be discarded. This leaves LoRa as the main candidate to deploy the access network. LoRa transceivers will be placed in each GTN-P station, responsible for sending the measured data to the LoRa gateway. As explained in [4], this gateway was implemented with a Raspberry Pi 3B+ in previous Antarctic campaigns of the SHETLAND-NET project, and it is responsible for storing the gathered data from all the sensors it is giving service to, ready to send all these data through the backbone network.

The backbone network will be composed of all NVIS nodes, which will interconnect remote WWSNs through the long backhaul links to form the GWSN. Each NVIS node mainly consists of a Red Pitaya, a Raspberry Pi 3B+, and an HF antenna [7]. The link that can be established between two NVIS nodes has a range of up to 250 km. In order to interconnect all the WWSNs and reach all remote areas, a multi-hop network will need to be deployed. Thus, some of the NVIS nodes will have to act as repeaters. At least one NVIS node will need to be connected to the control center in order to send all the data to it. To avoid a single point of failure (SPF), having more than one NVIS node connected to the control center is recommended. The possibility of having multiple paths to reach one destination demands the need for a routing protocol able to find the best possible loop-free path in the network [28].

The operation of the backbone network can be summarized as follows. Each NVIS node will act as a concentrator, gathering the data from every GTN-P station inside their LoRa coverage area. Once all possible data are collected, the NVIS node will forward it to the node connected to the control center, following the path determined by the routing protocol through the backbone network. 

However, we can find three main issues that can provoke this architecture to become a challenging network [14]:

1. Due to the fact of Antarctica’s extreme weather and environmental conditions, both sensors and gateways could experience temporary or persistent malfunctioning;
2. The irregular elevations of the Antarctic terrain might create situations where sensors do not have a LoS path through the gateway. This fact degrades the performance of LoRa communications considerably [23];
3. Depending on the ionosphere’s state and the solar activity, NVIS links may become unavailable temporally or intermittently [4,6,7,11].

For this reason, our primary goal was to establish a model to measure the trustworthiness of a CPS, with which the performance of the proposed architecture can be evaluated and its weaknesses detected and improved. Our model will be used in the pre-deployment phase of the SHETLAND-NET project to foresee performance difficulties of the defined architecture that may arise during its deployment in the field and predict the effect of the proposed countermeasures.

## 5. Trustworthiness Model Definition

**Figure 2:**
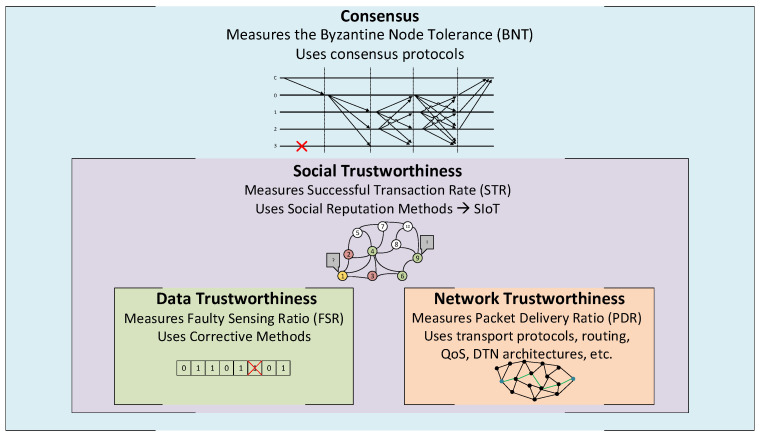
Layers of the proposed trustworthiness model.

Our model proposal to measure the trustworthiness and evaluate a CPS’s performance (in our case, a group of interconnected remote Antarctic wireless sensor networks providing an IoT telemetry service) is a layer-based model. This model is characterized by two base layers (Data Trustworthiness Layer and Network Trustworthiness Layer), two extension layers (Social Trustworthiness Layer and Consensus Layer), and the interaction among all of them. The Data Trustworthiness, Network Trustworthiness, Social Trustworthiness, and Consensus Layers can collectively define a system’s trustworthiness. A graphic representation of our layered model is shown in Figure 2. 

We postulated that each layer is characterized by its definition (scope), how the trustworthiness of that layer is measured (metric), and how the value of this metric can be improved (correction). 

On the one hand, Data and Network Trustworthiness are the base layers of our model, because the system that we want to measure is meaningless if we do not have data to be exchanged between nodes through a network. On the other hand, Social Trustworthiness and Consensus are the extension layers because they include functionalities that are not needed in the service architecture but are optional to implement.

### 5.1. Trustworthiness Layers’ Definitions

We propose the following definitions for each layer, based on the classification of trustworthiness approaches we defined in Section 3:

1. Data Trustworthiness Layer: Is the layer responsible for ascertaining the correctness of the data provided by the source;
2. Network Trustworthiness Layer: Is the layer responsible for assuring that a packet reaches its destination on time and unaltered despite the adversities (e.g., link failure, link saturation, or malicious attacks);
3. Social Trustworthiness Layer: Is the layer responsible for leveraging the capability of the objects to establish social relationships autonomously between them to improve the trust between them and the correctness of gathered data;
4. Consensus Layer: Is the layer responsible for reaching a state where all participants of a group agree on the same response or result.

### 5.2. Trustworthiness Layers’ Metrics

Managing the trustworthiness of a system is possible when the different layers are separately understood. This way, objectives and metrics can be defined to measure the level of trustworthiness. In order to measure the four layers of trustworthiness, we have defined a quantitative metric for each layer. Once metrics are defined, a trustworthiness target can be determined, which is a quantitative objective given to a trustworthiness metric. If a trustworthiness characteristic does not meet its target, a change factor is needed to revert the situation. The combination of all change factors defined to meet the trustworthiness targets is called the trustworthiness implementation.

We propose that the trustworthiness model will use the normalized metrics defined in Table 2 to quantify and measure trustworthiness. We selected these well-known metrics as they are also used to measure the impact of the technologies and approaches described in Section 3. We normalized all of them for better cohesion with our layer-based approach.

The faulty sensing ratio (FSR) is defined as the proportion of false sensed values (*FSV*) by all nodes and total sensed values (*TSV*) in a defined time period as stated in Equation (1).

(1) $$\mathrm{FSR}=\frac{FSV}{TSV}\;.$$

We considered that a sensed value is every independent and semantically significant measured data that a sensor stores in its memory (e.g., RAM, Flash, hard drive). Suppose no corrective methods are used in the system. In that case, sensed data (e.g., temperature, humidity, position, ice content) are considered to be faultily sensed if the value stored in the sensing (source) node’s memory is different from the value that the sensor should have correctly read (within a tolerance percentage). In real implementations, the number of FSV can only be measured if the sensed data’s value is known a priori (ground truth) [61]. Otherwise, only in simulations it is possible to quantify the number of FSV. FSV and TSV are parameters that must be gathered within the same time slot to calculate the ratio correctly. The lower the FSR, the better the data trustworthiness.

The packet delivery ratio (PDR) is calculated as the quotient between the total number of packets received (*Pr*) by all nodes and the total number of packets sent (*Ps*) by all nodes in the same time slot as stated in Equation (2). A packet is considered received if and only if the reception time (T_rx_) is less or equal to the transmission time (T_tx_) plus a defined threshold offset η (T_rx_ ≤ T_tx_ + η), and the packet content is not altered. The higher the PDR is, the better the network trustworthiness. In our proposal, retransmitted packets (if any) and original packets are counted separately to compute the metric value.

(2) $$\mathrm{PDR}=\frac{Pr}{Ps}\;\;.$$

The successful transaction rate (STR) is the proportion between the number of successful transactions (STs) and the total number of transactions (TTs) in a defined time slot as stated in Equation (3). We defined a transaction, *l*, as a sensed value, *v*, that a node, *j*, expects to receive from a node or group of nodes, *i*. Retransmitted or duplicated packets for the same value, *v*, are considered part of a single transaction, *l*. A transaction, *l*, is considered successful when a node, *j*, expects to get some information or data (*v*) from node *i* before a defined maximum reception time (Trx_max_) and receives it as expected, thus providing good feedback (*f_ij_^l^* = 1) for that transaction to node *i*. *ST* and *TT* are parameters that must be gathered within the same time slot to calculate the ratio correctly. The higher the STR, the better the social trustworthiness.

(3) $$\mathrm{STR}=\frac{ST}{TT}\;.$$

The byzantine node tolerance (BNT) is defined as the proportion of the supported byzantine nodes (*Nb*) that can participate in the consensus system without affecting the correctness of the general agreement and the total number of nodes (*Nt*) that participate in the consensus system as stated in Equation (3). A node is considered to be byzantine if it experiences a crash or soft fault that incapacitates it to behave as expected, or if it does not behave as expected on purpose (malicious node). The higher the BNT, the higher the probability to reach a correct general agreement. Although theoretically, the BNT value range is between 0 and 1, in practice, it is not possible to reach a correct consensus with a BNT ≥ 0.5.

(4) $$\mathrm{BNT}=\frac{Nb}{Nt}\;.$$

### 5.3. Trustworthiness Improvement Examples

Now that we have defined the four trustworthiness layers and their associated metrics, we can give some examples of techniques and protocols that can be used as countermeasures at each layer to improve the metrics’ values. 

#### 5.3.1. Data Trustworthiness Countermeasures 

At the Data Trustworthiness Layer, corrective methods can be applied that try to detect abnormal data (false sensed values) stored in the source node due to the fact of a sensor malfunctioning, a misreading of the sensed data, or erratic writing to the node’s memory. Corrective methods can be used to detect and correct these abnormal values by comparing them to the values sensed by the same node previously and other mechanisms such as hashes, checksums, and parity bits. If these corrections are performed at the post-processing stage by the receiving server or gateway, the nodes’ malicious data manipulation can also be detected. However, our model assumes that corrections are only made by the own node (source node). Otherwise, errors that originated during the data transport through the network, which are out of our scope of definition for the Data Trustworthiness Layer, could be misinterpreted as source node errors. The drawback of this assumption is that only non-malicious errors are likely to be corrected at this layer because malicious nodes might not correct data on purpose. Our model specifies that other layers of the model are responsible for mitigating malicious behaviors (e.g., the Network Trustworthiness Layer).

The method presented in [35] is a suitable example of a corrective method for data trustworthiness. This value-level corrective method defines thresholds to detect potential abnormal data (e.g., a lower-value limit t_low_, an upper-value limit t_up_, and an abrupt change threshold t_ch_). When a potential abnormal value is detected, it is compared with the values sensed from the node’s neighbors, computing the group value similarity (G). Since this breaks our model’s assumption, this value similarity could be computed with the historical values from the sensor itself as in [36]. If the similarity is lower than a threshold t_sim_, then the abnormal data are confirmed and corrected (e.g., interpolation with previous and posterior correct values sensed by the own node). This method might experience false positives (by detecting a correct value as abnormal and modifying it) and false negatives (by not detecting an abnormal value), which can be grouped into faulty sensed values (FSV). If the thresholds are too strict, the number of false positives will increase, while the number of false negatives increases if the thresholds are too lax. The fewer the number of FSV, the better the data trustworthiness, so an optimal trade-off value for the thresholds must be found to minimize the overall number of FSV. This number is easy to gather in simulation scenarios, but in real implementations, it is only possible if the values are well-known a priori (ground truth values).

#### 5.3.2. Network Trustworthiness Countermeasures

At the Network Trustworthiness Layer, transmission coding techniques such as FEC convolutional codes [38], LDPC codes [39,40], and polar codes [41] are used to increase the robustness of the transmitted signal. Routing protocols and quality of service (QoS) mechanisms are used to find the best path from a source to a destination by quantifying the quality or performance of each link in the network. For each destination, more than one path can be determined as feasible thus providing load balancing. Many metrics exist to calculate the best path such as the number of hops, the bandwidth of the link, the delay, and the expected number of retransmissions. These routing protocols can be classified under different categories such as proactive/reactive, link-state/distance-vector, or monometric/multimetric [45]. Selecting the best path for a traffic flow will eventually improve network statistics such as throughput, delay, jitter, or packet delivery ratio (PDR). In the case of challenging networks, DTN overlay architectures and protocols, such as the Bundle Protocol [62], is also a solution that can be used to improve the network trustworthiness.

Another relevant element to take into account in this layer is the data security through the network. While traveling from the source to the destination, data should remain private, available, and unaltered, preventing it from cyberattacks [63]. For this purpose, network elements such as next-generation firewalls or intrusion detection systems and security mechanisms, such as data encryption, authentication, anti-spoofing techniques, and network filters, are used in the network.

#### 5.3.3. Social Trustworthiness Countermeasures

At the Social Trustworthiness Layer, most solutions tend to use reputational mechanisms to determine which nodes to trust when exchanging information. This reputation is commonly based on previous transactions’ feedback to build an opinion for the node’s trustworthiness [64]. More complex and robust mechanisms also incorporate parameters such as the indirect opinion of other nodes, the relevance (weight) of each transaction, the node’s centrality, the node’s computational capacity, and the type of relationships between the nodes [50]. 

The model in [51] provides two different ways for computing the reputation of a node. On the one hand, a subjective model of social trustworthiness is presented to compute the reputation of node *i* under the perspective of every other node (*R_ij_*), these reputations being different from each other, because the experience of interaction with node *i* for two different nodes can be different. Moreover, reputations are asymmetric, meaning that the reputation that node *j* calculates from node *i* can be different from the reputation that node *i* calculates for node *j* (*R_ij_* ≠ *R_ji_*). Thus, the system’s overall trustworthiness can be represented as an N × N matrix for the reputation that each node calculates for all the other nodes, where N is the total number of nodes. On the other hand, objective models calculate one single reputation for each node (*R_i_*), representing the trustworthiness that the system as a whole perceives from node *i*. This reputation takes into account the opinion and the feedback from all the other nodes. Thus, the system’s overall trustworthiness is represented as an N-size vector with the reputation that the whole network perceives for each node.

Both the subjective and objective approaches aim to leverage the transactions between trustful nodes and isolate those with bad reputations, which are considered more faulty or malicious prone. Thus, their goal is to maximize the number of successful transactions (STs). 

#### 5.3.4. Consensus Countermeasures

At the Consensus Layer, several mechanisms can be used to reach a decentralized general agreement (GA) that all nodes in the group consider to be true. Theoretically, if the number of byzantine nodes is more than 50% of the total number of participating nodes, every consensus mechanism will fail to reach a benevolent agreement. Consensus mechanisms aim to reach the GA while tolerating a percentage of byzantine nodes. Consensus protocols are generally classified into competing mechanisms (proof-based) and voting-based mechanisms. The latter are more suitable for IoT devices because they consume fewer resources from the node. These protocols commonly consist of various voting phases to reach the GA, and their goal is to maximize the number of tolerated byzantine nodes (BNs). A drawback of these mechanisms is that they need participating nodes to exchange a large number of messages between them to reach a consensus which can be a problem in low-bandwidth networks, consuming most of this bandwidth. Some protocols look for a trade-off between the number of tolerated BNs, throughput, and scalability.

### 5.4. Trustworthiness Layers’ Dependencies

**Figure 3:**
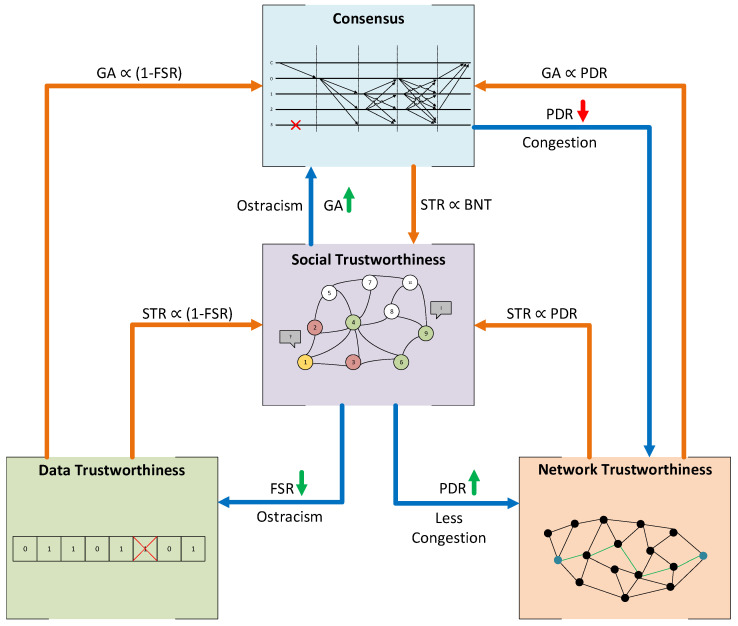
Dependency diagram between trustworthiness layers.

Trustworthiness layers’ dependencies must also be understood before deploying the system’s architecture. In this way, we can build more accurate expectations of how the model’s overall trustworthiness and concrete layers will be affected by applying a trustworthiness countermeasure in one layer. If the impact of applying specific countermeasures could not be foreseen, their implementation in the field could negatively affect the overall system’s trustworthiness. For instance, how will the data trustworthiness affect the consensus? Can a robust consensus protocol lower the trustworthiness of the network because it is causing bottleneck congestion? In the SHETLAND-NET project, the trade-offs between these layers need to be carefully analyzed before deploying the system in the field to obtain the optimal overall trustworthiness level. If we were not considering these dependencies, it could be possible to experience a degraded performance of the deployed architecture without the necessary resources or response time to correct it during the campaign. Our model dependencies proposal is exhibited in Figure 3. These dependencies are qualitatively analyzed below, and the simulation tests performed in Section 6 were necessary to validate the model and quantify their actual impact on the overall system’s trustworthiness.

The Consensus Layer is directly affected by the other three layers. If FSV (Data Layer) is closer to 0, it means that nodes tend to measure the sensed values correctly, so they will be more prone to reach a correct general agreement. From the Social Layer, it is possible to ostracize those nodes with a lower reputation (which should be the ones with more false sensed values) if the application can afford to lose the data from them. In this case, if nodes with the worst reputation were omitted, it should be more probable to reach a correct general agreement for the rest of the nodes. Finally, suppose the PDR (network trustworthiness) is closer to 1. In that case, it means that the whole network delivers the most packets unaltered and on time, so fewer nodes will be considered byzantine due to the network issues and reaching correct general agreements will be more feasible. It is important to notice that all these dependencies do not affect the Consensus Layer metric, the byzantine node tolerance, which depends only on the consensus algorithm used and the total number of nodes participating in the consensus group.

We propose that the Social Layer can also be directly affected by the other layers. On the one hand, FSV and STR are inversely related. If the FSV is close to 0, a transaction coming from that node is less probable to have a false sensed value, meaning that it will become a successful transaction if the network delivers it properly to the destination. In addition, the source node will obtain good feedback from the receiving node, increasing its reputation. On the other hand, PDR and STR are directly related. As the PDR decreases, it is more feasible that packets targeted to a node are lost in the network, decreasing the STR. Thus, the receiver would evaluate the transaction as a failure, providing bad feedback and decreasing the sender’s reputation. Finally, if the Consensus Layer is implemented, the negative effect of some false sensed values from byzantine nodes and lost packets can be masked thanks to the consensus algorithm. Nodes could still reach a correct general agreement, marking that transaction as successful and increasing the STR.

The network layer can be directly affected by the Social and Consensus Layers in terms of congestion [65,66]. Depending on the application, if nodes with the lowest reputation could be ostracized, their sensed data might not be sent through the network because they might not be requested. Thus, these nodes’ links might be less congested and less prone to packet drops, increasing the PDR. Adversely, as mentioned before, using a consensus mechanism introduces a considerable amount of network traffic. In addition, the number of messages exchanged between a group of nodes is directly proportional to the number of nodes in the group. Thus, if the network bandwidth was not enough to support this extra traffic, the network could be more prone to be congested and drop packets, decreasing the PDR.

Finally, it is intuitive to think that the Data Layer should not be affected by the other layers. The variability of the FSV should depend on the error probability of the sensors and the node itself (e.g., equipment quality, battery degradation), which could also be affected by external factors (e.g., environmental characteristics). However, we propose that the Data Layer can be affected by the Social Layer. Suppose the Social Layer is implemented and is being used to ostracize the lowest reputation nodes. In that case, we considered that sensed values from omitted nodes must not be counted for the FSR computation. Thus, if the lowest reputation nodes were the ones with more false sensed values, the overall FSR should increase.

It is important to see that Data and Network Layers (the base layers, which are always present) are entirely independent, given that the correctness of data is always measured on the source node, never on the destination. This way, data loss or alteration caused by the network does not affect the data correctness measure.

Notice that Social and Consensus Layers (the extension layers, which are optional) are the ones affected by the rest of the layers. However, the way they are affected is different. On the one hand, the dependencies from other layers to the Social Layer directly affect the value of its trustworthiness metric, the STR. On the other hand, the Consensus Layer metric, the BNT, is not affected by other layers, but these dependencies can improve the probability of reaching a correct general agreement, which in final terms improves the Social Layer metric, the STR. 

In that sense, we considered that the system’s overall trustworthiness can be measured with the STR metric, which is the one affected by the four layers of our model, and intrinsically incorporates the effects of the other three metric values (i.e., FSR, PDR, and BNT). Moreover, notice that without implementing the extension layers, the STR can still be computed, which will combine the effects of the base layers (i.e., Data and Network Trustworthiness).

Although the dependencies between the layers and metrics of our model have been identified, it is still challenging to quantify the effect of looped dependencies on the system’s trustworthiness. We identified two actions that can provoke a direct looped dependency. First, if Social Trustworthiness Layer is used to ostracize the lowest reputation nodes, their sensed values will be omitted, decreasing the FSR and eventually increasing the STR. However, suppose more traffic than supported by the network is concentrated on the links that lead to most reputation nodes. In that case, it is possible to create network congestion that will decrease the PDR and eventually decrease the STR. Second, implementing a consensus mechanism might help tolerate byzantine nodes and faulty network links, which eventually increases the STR. Nonetheless, suppose the network bandwidth is not large enough to allocate the extra traffic introduced by the consensus mechanism. In that case, the network may suffer from congestion, decreasing its PDR and eventually decreasing the STR.

To quantify the effects and trade-off points between these dependencies, it is essential to test the model’s applicability with a use case and measure the trustworthiness metrics under different circumstances and several times. Given the complexity and cost of performing such a number of tests in the field during the Antarctic campaign, we opted to use simulation tests, which provides more flexibility. These pre-deployment simulations will help us decide which are the most suitable and trustworthy architectures for our system and anticipate the possible weaknesses and problems that may arise during the deployment in the field.

## 6. Simulation Tests

To validate the trustworthiness model, it was necessary to measure the metrics values for the use case scenario several times under different circumstances. For this purpose, the use case scenario was represented and evaluated in the Riverbed Modeler Simulator [19]. As stated before, our use case scenario was a group of interconnected remote Antarctic wireless sensor networks providing an IoT telemetry service. Concretely, the telemetry service will be used to automatize the data gathering of GTN-P stations to study the permafrost of the Antarctic region. The remote sensors of WSNs will be connected to a concentrator gateway through LoRa (access network), and these gateways will be interconnected between them and a control center through long backhaul NVIS links (backbone network). The extreme conditions GTN-P stations need to work with, added to the challenges of NVIS links and a LoRa network without LoS, might degrade the overall system’s trustworthiness. In order to foresee which problems may occur during the Antarctic campaign and build more accurate expectations of the system’s performance and outcomes, we applied our proposed trustworthiness model to measure and evaluate them.

The first step was the modeling of the network, the nodes, and the application. Once the model is designed and implemented in the simulator, the set of tests and the simulation parameters must be defined. After that, the simulations were run, and results were collected and evaluated.

### 6.1. Network Models

For the use case scenario, the backbone network (NVIS) and the access network (LoRa) were modeled separately. On the one hand, the NVIS channel was modeled following the characteristics described in [7]. The transmission frequency was 4.3 MHz with a channel bandwidth of 2.3 kHz and a bit rate of 4.6 kbps. An FEC convolutional coding with a ½ rate code and interleaving were used to increase the reliability of the transmission. The range of the HF link was up to 250 km. 

**Figure 4:**
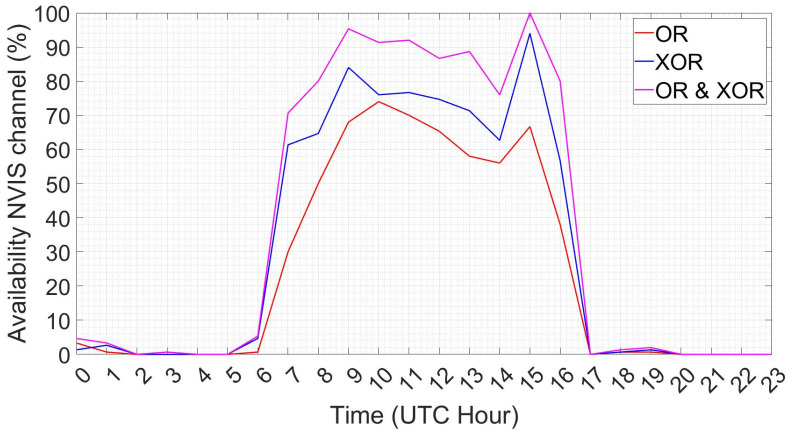
NVIS link availability depending on solar activity and the ionosphere’s state [11]. The graph’s legend is defined as follows: OR refers to the performance of the ordinary wave, XOR refers to the performance of the extraordinary wave received. OR and XOR refer to the total performance between both the ordinary and extraordinary modes. We have the copyright of [11], it belongs to the GRITS research group from La Salle.

Moreover, given that the ionosphere characteristics vary considerably throughout a day, we also modeled the probability of a packet being correctly delivered through an NVIS link hour by hour, following the results in [11]. These results showed that the NVIS links are unlikely to be available from 17:00 until 6:00. In contrast, the channel availability from 6:00 to 17:00 varied from 70% to 100% when both the ordinary and extraordinary waves received were combined as shown in Figure 4.

On the other hand, the LoRa channel was modeled based on the results shown in [23,67]. The transmission frequency was 868 MHz with a channel bandwidth that varied depending on the chosen data rate (DR) and the rate spreading factor (SF). In our case, we chose CR3 (4/7) and SF7, resulting in a channel bandwidth of 125 kHz and a bit rate of 5.47 kbps. The range of the link was up to 30 km. In the line of sight (LoS) case, the channel was always available with a packet loss of 0%. Otherwise, with no LoS, the packet loss varied from 0% to 98% depending on the signal reflections, with an average value of 72%. Due to the Antarctic surface’s irregularities, we cannot assume that the GTN-P stations will be located in LoS with the gateway. For this reason, we considered that 25% of the sensors will have a LoS to the LoRa gateway, while the remaining 75% will not have LoS. Table 3 summarizes the characteristics of our network models.

### 6.2. Node Model

In the case of the node, both the GTN-P station and the gateway will use the same finite state machine model. The “INIT” state initializes the model and its attributes. The “IDLE” state is used when the node is waiting for a packet to arrive, transitioning to the “PROCESS” state, or a self-interruption to send the sensed data values, transitioning to the “SEND” state. 

### 6.3. Application Model 

Pseudocode algorithms for the application modeling are shown in Appendix A, Algorithms A1, A2, and A3. The application consists of the telemetry service to gather data from measured values by sensors and send them to the control center. To better understand the below explanations, we encourage revisiting Figure 1 to recall the proposed network architecture.

Each measured value, *v*, is considered a transaction, *l*, that must reach the control center. The application can be run without implementing any of the extension layers of the proposed trustworthiness model (standard mode) or can implement the Social or Consensus Layers of the model (redundancy mode), inclusively or exclusively. In standard mode, each value, *v*, is measured by a single GTN-P station, while in redundancy mode, the implementation of a reputational or consensus mechanism leverages the creation of clusters (groups of GTN-P stations) that measure the same value *v*. 

The GTN-P stations will send data packets once per hour, simulating the moment when the 32 values are gathered from the GTN-P station sensors, stored in memory, and delivered to the gateway. We decided to sense these values hourly because it is the same sensing frequency that the members of the PERMASNOW project [18] used when they performed their automatization tests. In this process, if no consensus mechanism is performed, a hardcoded value, *v*, for each parameter will be inserted into a 132 byte payload (32 values and a timestamp, 4 bytes each). With a probability, *Pb*, the value, *v*, will be modified to another value out of an acceptable range (*v_min_*, *v_max_*), and the total number of FSV will be increased by one. This payload will then be inserted into the packet to be sent to the gateway. If an ACK packet is not received from the gateway before a timeout *T_out_*, the data packet will be retransmitted up to a maximum of three times. In the case of implementing a consensus mechanism, all the GTN-P stations participating in the cluster (which are measuring the same *v*) will start the process to reach a general agreement. Once they have reached it, only the cluster leader will send the payload to the gateway with the agreed value *v*. During the consensus process, if the Social Layer is also implemented, each packet exchanged between the nodes participating in the consensus group will be used to compute the reputation *Ri* of nodes. The node with the highest reputation will be elected as the group leader. Moreover, a node *i* with a reputation *R_i_* lower than *R_min_* will not have the right to vote for the value election. However, it will be allowed to continue participating in the consensus group to increase its reputation until it can be granted the right to vote again. On the contrary, if no reputational mechanism is being used, all group members will always have the right to vote, and the leader will be chosen randomly.

On the other site, gateways will collect the data from the GTN-P stations inside its LoRa coverage area and then forward it through the NVIS backbone network until it reaches the control center. Given that gateways are also nodes, they may experience a byzantine failure with probability *Pb*. In that case, the gateway will modify the payload’s content. In standard mode, each value *v* received from node *i* must be forwarded to the control center. In redundancy mode, if no consensus mechanism is being used (only the Social Layer is implemented), the gateway will receive several candidates for the value *v* from every node in the cluster. The gateway will inspect the values from it and check if they are in the acceptable range (*v_min_*, *v_max_*). In an affirmative case, gateway *j* will provide positive feedback for that transaction *l* from node *i* (*f_ij_^l^* = 1). Otherwise, the feedback will be negative (*f_ij_^l^* = 0). After providing feedback for every transaction, the reputation *Ri* of the nodes will be updated, and the value provided from the node with the greatest reputation will be chosen as the definitive value *v*. Alternatively, if a consensus mechanism is used in redundancy mode, the gateway will only receive a single value *v* for each cluster, which will have to be forwarded to the gateway.

Due to the NVIS backbone network’s unavailability during night hours (from 17:00 until 6:00), values received by the gateway during this period will be stored in the gateway’s memory and forwarded to the control center later (when the NVIS links start functioning at 6:00). On the contrary, values received during daytime (from 6:00 until 17:00) will be forwarded to the control center as soon as the gateway receives and processes them. As GTN-P stations do, the gateways also expect to receive an ACK packet for every payload packet they send to the control center. If an ACK packet is not received from the control center before a timeout, *T_out_*, the data packet will be retransmitted up to a maximum of three times.

Finally, the control center will receive all the transactions that had not been lost through the network. Each value *v* from the received payload by the control center from node or node cluster *i* will be considered a transaction *l*. The control center will compute the STR by comparing the received values for each payload with the hardcoded values.

The probability, *Pb*, of a node having a byzantine fault is unlikely to be constant over time. As stated in [68], by associating the battery discharge to the WSN node aging process, the node reliability can be identified and associated with the battery charge level. Thus, following the model in [69], we can assume the impact of aging following a linear form as defined in Equation (5):

(5) $$Pb\left(t\right)=Pb_{0}+kt,$$

where $Pb_{0}$ is the probability of a node having a byzantine fault at time t=0, and k is the aging factor. Thus, the probability of a node having a byzantine fault will increase hour by hour until its battery is completely drained at $t=t_{d}$, when it experiences a crash fault and $Pb\left(t_{d}\right)=1$. However, this model will only be applied to GTN-P stations that will be powered by batteries. On the contrary, we assume that the gateways will always have a constant power supply in our use case because they will be placed in the research base. Thus, their probability of experiencing a byzantine fault will remain constant over time as defined in Equation (6):

(6) $$Pb\left(t\right)=Pb_{0}.$$

As explained in Section 4, the use of corrective methods to improve the data trustworthiness provoke, in practice, that the probability, $Pb_{0}$, of a node experiencing a byzantine fault will decrease, thus reducing the number of FSV. For that reason, different values of $Pb_{0}$ will be used in our simulations to emulate the use of different corrective methods.

### 6.4. Social Trustworthiness Model

The reputational model for implementing social trustworthiness in our use case is a simplified version of the objective model defined in [51]. Our use case simplification assumes that all transactions will have the same weight, all nodes have the same computational capability, and the relationship factors between them are equal. Thus, the reputation $R_{i}$ of node *i* can be measured as defined in Equation (7):

(7) $$R_{i}=\alpha O_{i}^{short}+\left(1-\alpha \right)O_{i}^{short}\;$$

where $O_{i}^{short}$ is the short-term opinion of node *i*, $O_{i}^{short}$ is the long-term opinion of node *i*, and α is a design value between (0, 1) to ponder the importance of short-term and long-term opinions. The short-term opinion of node *i* is measured as stated in Equation (8):

(8) $$O_{i}^{short}=\overset{M}{\underset{j=1}{\sum }}\overset{L^{short}}{\underset{l=1}{\sum }}C_{ij}f_{ij}^{l}/\overset{M}{\underset{j=1}{\sum }}\overset{L^{short}}{\underset{l=1}{\sum }}C_{ij},\;$$

where M is the total number of nodes of the group, excluding node *i*, $L^{short}$ is the number of last *l* transactions considered to be relevant for building the short-term opinion, $f_{ij}^{l}$ is the feedback that node *j* gave to node *i* for transaction *l*, and $C_{ij}$ is the credibility of node *j* to evaluate node *i*. 

Analogously, the long-term opinion is calculated as defined in Equation (9):

(9) $$O_{i}^{long}=\sum_{j=1}^{M}\sum_{l=1}^{L^{long}}C_{ij}f_{ij}^{l}/\sum_{j=1}^{M}\sum_{l=1}^{L^{long}}C_{ij}\;,$$

where $L^{long}$ is the number of last *l* transactions considered to be relevant for building the long-term opinion, and $L^{long}>L^{short}\;$. The credibility of node *j* to evaluate node *i* is calculated as shown in Equation (10):

(10) $$C_{ij}=\frac{R_{j}}{1+\log\left(N_{ij}+1\right)}\;,$$

where $N_{ij}$ is the number of transactions between node *j* and node *i*.

### 6.5. Consensus Model

A consensus protocol can be modeled by knowing the background traffic (bps) that is introduced into the network and the number of byzantine nodes supported (Nb). In our use case, each group of redundant GTN-P stations will run the practical byzantine fault tolerance (PBFT) algorithm [70]. From [71], we can assume that the background traffic exponentially grows as the number of nodes participating in the consensus (Nt) group increases. Moreover, the number of tolerated byzantine nodes, Nb, is calculated as:

(11) $$Nb=\frac{Nt-1}{3}\;\;,$$

In the simulation, if more than Nb nodes experience a byzantine behavior, the agreement reached will have incorrect values. Otherwise, the resulting payload will contain the correct values.

### 6.6. Tests Definitions

A summary of the characteristics of the simulation tests is shown in Table 4.

Each different test will be run 30 times, which gives us the total amount of 113,400 tests. Each test has a simulation duration of 5 days (120 hours), and the average value of the STR trustworthiness metric will be calculated. The different byzantine probabilities are proposed to simulate scenarios with different corrective methods that can reduce the byzantine probability of a node. On the other hand, two different routing protocols will be used to analyze which kind of method (reactive or proactive) has a better impact on the service’s trustworthiness. Moreover, the Consensus and Social Trustworthiness Layers will be implemented or not to analyze their influence on the service’s performance. Finally, the impact of the number of nodes connected to each gateway will also be studied by varying them. In standard mode, there was only one GTN-station per group (because there is no redundancy). This means that each of the 9 $Pb_{0}$ values must be tested against each number of GTN-P clusters per gateway, giving us a total of 90 possibilities. In redundancy mode, this number increases to 900 since the number of GTN-P stations per cluster varies from 1 to 10. If we sum the cases of standard mode, redundancy mode with consensus, and redundancy mode with social trustworthiness, we have a total of 1890 different cases, which are doubled to 3780 considering that we want to test the system with two different routing protocols. Considering that each test is repeated 30 times, a total of 113,400 simulations were run in the simulator.

## 7. Simulation Results

**Figure 5:**
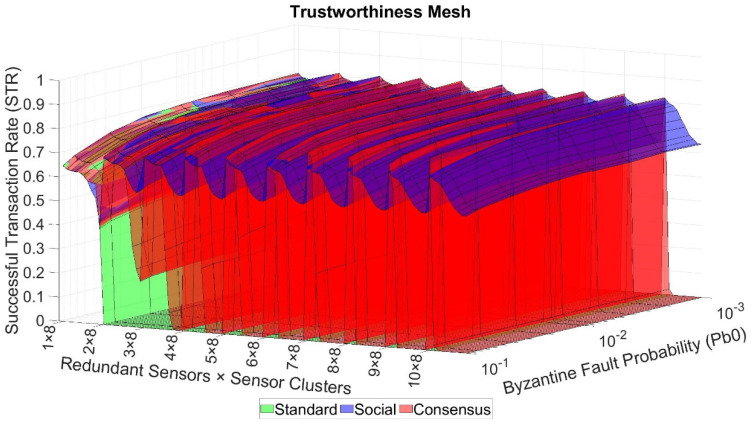
Trustworthiness mesh graph for the standard operational mode (green), the redundancy mode with Social Trustworthiness (blue), and the redundancy mode with Consensus mechanism (red).

**Figure 6:**
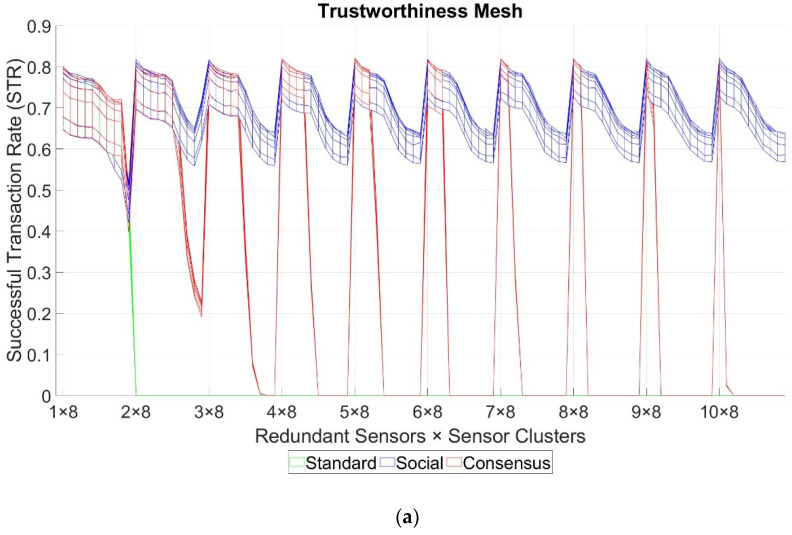

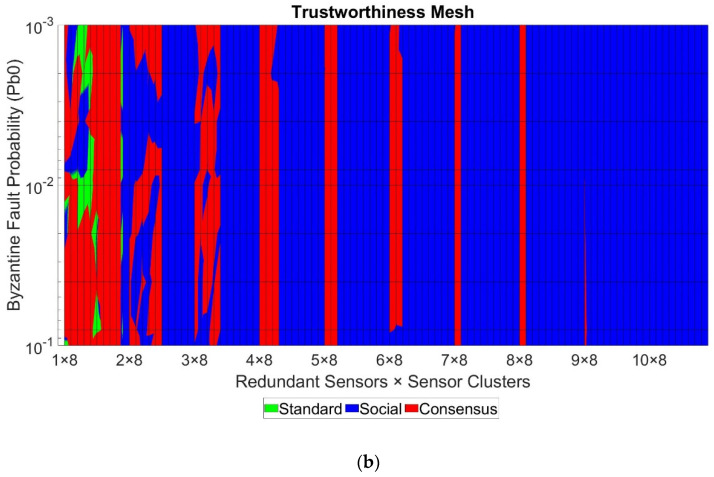
Two-dimensional views of the trustworthiness mesh graph for the standard operational mode (green), the redundancy mode with Social Trustworthiness (blue), and the redundancy mode with consensus mechanism (red): (**a**) Frontal view of the trustworthiness mesh (STR vs. number of nodes); (**b**) top view of the trustworthiness mesh (byzantine fault probability vs. number of nodes).

After performing all the simulations, the average value of the STR was calculated for every set of 30 runs per test. The obtained results had a maximum error deviation of 0.61% with a confidence interval of 99%. Three different operational modes for the telemetry service can be clearly identified: the standard mode, the redundancy mode with Social Trustworthiness Layer, and the redundancy mode with Consensus Layer. For every mode, an N x M-dimension grid with all the possible combinations of stimulation parameters was formed, where M is the number of different $Pb_{0}$ values (nine in our case as stated in Table 4, row 5), and N is the number of different GTN-P node combinations per gateway (10 in standard mode and 100 in redundancy mode). For every point in this grid, the average value of the trustworthiness STR metric was computed. If we link all the STR values for every neighboring point in the grid, a mesh with all the STR values is formed. We call this mesh the trustworthiness mesh. Figure 5 exhibits the trustworthiness mesh three-dimensional graph for all the operational modes. Given that the differences between the AODV and OLSR scenarios’ obtained results are negligible, only the results for the AODV scenarios are shown. Figure 6 shows different two-dimensional perspectives of the trustworthiness mesh graph to understand and analyze the results better. For both figures, the “byzantine fault probability” axis has nine discrete points, which are (1 × 10^−3^, 2 × 10^−3^, 4 × 10^−3^, 8 × 10^−3^, 1 × 10^−2^, 2 × 10^−2^, 4 × 10^−2^, 8 × 10^−2^, 1 × 10^−1^). The “redundant sensors x sensor clusters” axis has 100 discrete points, according to Table 4, rows 11 and 12, which are (1 × 2^N^, 2 × 2^N^, …, 10 × 2^N^), where N = (3, 4, …, 12).

From Figure 5 and Figure 6, we can analyze the behavior of the trustworthiness mesh. We can see how without redundancy, the STR is always lower than 0.8. Thus, we can conclude that due to the characteristics of the NVIS and LoRa networks, the threshold of maximum trustworthiness that can be achieved is approximately 80% of total transactions in our use case. This means that, on average, each monitored value arrives at the control center 19 times a day at most. The authors of [18] left the complete automatization of this use case as an open challenge, aiming to increase the monitoring frequency to visualize the daily variations of the monitored properties (air, snow, bottom snow, surface, and ground temperature, among others). Our project’s objective was to receive 14 out of 24 (58.33%) values each day at least. This is the minimum acceptable threshold to achieve the goals in [18]. In the tests results, acceptable STR values (>0.58) are maintained if the number of sensor nodes is kept under 512, although it decreases below the desired trustworthiness threshold if the number of sensors per gateway is higher. Also, we can notice that the shape of the trustworthiness mesh is practically identical for all three cases in the “1 × N sensors” zone (no redundancy). This means that, as was expected, adding the Social or the Consensus Layers did not improve the level of trustworthiness if there was no redundancy.

From Figure 5 and Figure 6a, we can conclude that adding sensor redundancy and implementing the extension layers of our model improved the trustworthiness of the system, given that STR values greater than 0.8 were achieved. In cases of low redundancy (“2 × N sensors” and “3 × N sensors”), implementing the consensus mechanism did not improve the trustworthiness of the system when compared to the Social Trustworthiness case (the STR values are very similar). This is because, with two or three redundant nodes, the number of byzantine nodes tolerated by the consensus mechanism was still 0. Starting with four redundant nodes (“4 × N sensors”), the consensus mechanism’s effects started to be noticed, achieving better STR values than the Social Trustworthiness case.

However, as the byzantine fault probability of the nodes decreases (meaning the FSR is lower), the difference between the STR values from the consensus mechanisms case and the Social Trustworthiness case becomes smaller. This means that implementing a consensus mechanism is more appropriate when the probability of the nodes experiencing byzantine behaviors is relatively high, and it is not necessary when this probability is low. In our cases, differences between STR values from both cases were not relevant as $Pb_{0}\leq 0.01$.

Moreover, the quantity of network traffic that the consensus mechanism adds, combined with the LoRa and NVIS networks’ low bandwidth, provokes low scalability for this solution. We can see that by looking at the evolution of the consensus trustworthiness mesh’s STR values (red). We notice that as the number of sensor clusters increase, the STR values decreases until it drops to 0. This is because the nodes generate more traffic than the network supports. Thus, the network is congested, and the PDR rapidly decreases. Furthermore, the higher the number of redundant sensors per cluster, the sooner the STR dropping point (network saturation) happens. This resolves one of the looped dependencies postulated in Section 5.4.

On the contrary, it seems that implementing a social trustworthiness approach is more robust to these variations. Even if it did not achieve the same levels of STR as the consensus mechanism case when the number of sensor clusters was low, its STR values never dropped below 0.55 (very close to our desired minimum trustworthiness threshold), even in the scenario with more sensors and worse FSR. It is clear that the trustworthiness of the social case was also affected when the number of sensor nodes increased (which implies more network load and lower PDR), but its STR did not drop drastically and could maintain acceptable values. Due to the fact of our use case’s modeling, the social trustworthiness implementation did not ostracize the nodes so that their sensed values were not collected, and the network load decreased. This is because in our simulations, each node had the same probability of experiencing a byzantine fault or sensing the value correctly, so ostracizing one of them could negatively affect the results. Thus, the behavior of the other looped dependency postulated in Section 5.4. remains uncertain.

From Figure 5, we can also conclude, as expected, that data trustworthiness had a direct affection on the overall system’s trustworthiness. In all cases, as the byzantine fault probability, $Pb_{0}$, increased (meaning that more values are faultily sensed, increasing the FSR), the STR decreased. 

Finally, Figure 6b shows for each of the 900 possible scenarios which is the most trustworthy option to implement the service. From this view, we can clearly see the robustness of the social trustworthiness case, showing how it gains ground as the number of sensors in the network increases. 

## 8. Conclusions

This paper continues the SHETLAND-NET project’s task to design a remote WSN for the Antarctic region using NVIS technology. The article focused on the use case of deploying a group of interconnected remote Antarctic wireless sensor networks providing an IoT telemetry service. A system and network architecture to implement the telemetry service was defined, using LoRa at the access network and NVIS long backhaul links at the backbone network. The extreme conditions remote sensors need to work with, added to the challenges of NVIS links and a LoRa network without LoS, can provoke a degradation of the overall system’s trustworthiness. In order to study the viability of the service to be implemented before its deployment in the field during the Antarctic campaign, and aiming to anticipate the possible challenges that may arise, we proposed a model to measure and evaluate the trustworthiness of the system proposed. This trustworthiness model consists of four layers (two base layers and two extension layers) that can affect the successful transaction rate (STR) trustworthiness metric.

The trustworthiness model and the system architecture were validated using the Riverbed Modeler simulator. The obtained results have a maximum error deviation of 0.61% with 99% of confidence. The results show that the defined system architecture can reach acceptable levels of STR (>0.58) in case a relatively low number of sensors are deployed, although it drops too much with a large number of sensors. Adding redundancy to the measured values with multiple sensors and applying a social reputational mechanism improves the robustness of the system’s trustworthiness, reaching higher STR values (>0.8) and never dropping below 0.55 even in high sensor-density scenarios. On the contrary, applying a consensus mechanism improves the system’s trustworthiness when a low number of sensors are deployed. However, the STR values abruptly decrease as the number of deployed sensors increases. 

**Figure 7:**
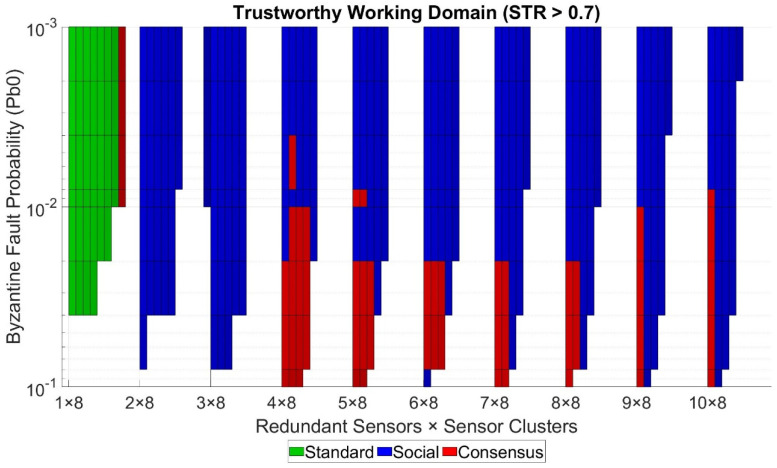
Trustworthiness mesh top view, coloring working domains with STR > 0.7.

Our model can also be used to visualize the work domain to implement our service, given a desired minimum trustworthiness level. For example, suppose our project requires a minimum STR of 0.7, so an average of 16 out of 24 sensed values per day reach the control center correctly. In this case, we could tolerate situations where the number of successful transactions that reached the control center was less than the average due to the fact of unexpected conditions but still achieving an STR higher than 58.33% to meet the objective of [18]. Figure 7 shows the work domain of our telemetry service for an STR higher than 0.7. For every point in the grid, if no solution provides an STR higher than the desired minimum value, the surface for that area is white-colored, meaning we cannot deploy the service with those conditions. On the contrary, if one or more solutions achieve an STR higher than the desired minimum value, the surface is painted with the color of the solution with the highest STR. However, it could be possible that we would prefer a solution that was not the one with the highest STR if all of the following criteria were met:

- The solution still has an STR greater than the desired minimum value;
- The solution has less cost than the solution with the highest trustworthiness in any sense (e.g., economic, computational resources, network load);
- The difference of the STR value achieved by the solution with less trustworthiness and the solution with the highest trustworthiness is less or equal to an established threshold value *d_max_*.

We first preferred to deploy the standard mode in our use case, followed by the redundancy mode with the Social Trustworthiness Layer implementation and the redundancy mode with Consensus Layer implementation. This way, we prioritized the solution with lower resource consumptions (computational and network loads). To construct Figure 7, we set the value of *d_max_* to 0.01. For our use case example, we chose this value arbitrarily. However, the value of this design parameter must be carefully analyzed for every particular use case to choose the actual optimal solution. The graph provides a clear vision of the work domains or areas that meet the necessary conditions to deploy the requested service guaranteeing the required minimum trustworthiness level. Furthermore, we can identify which solution to implement with the highest STR or the best trade-off between cost and STR for every grid point.

**Figure 8:**
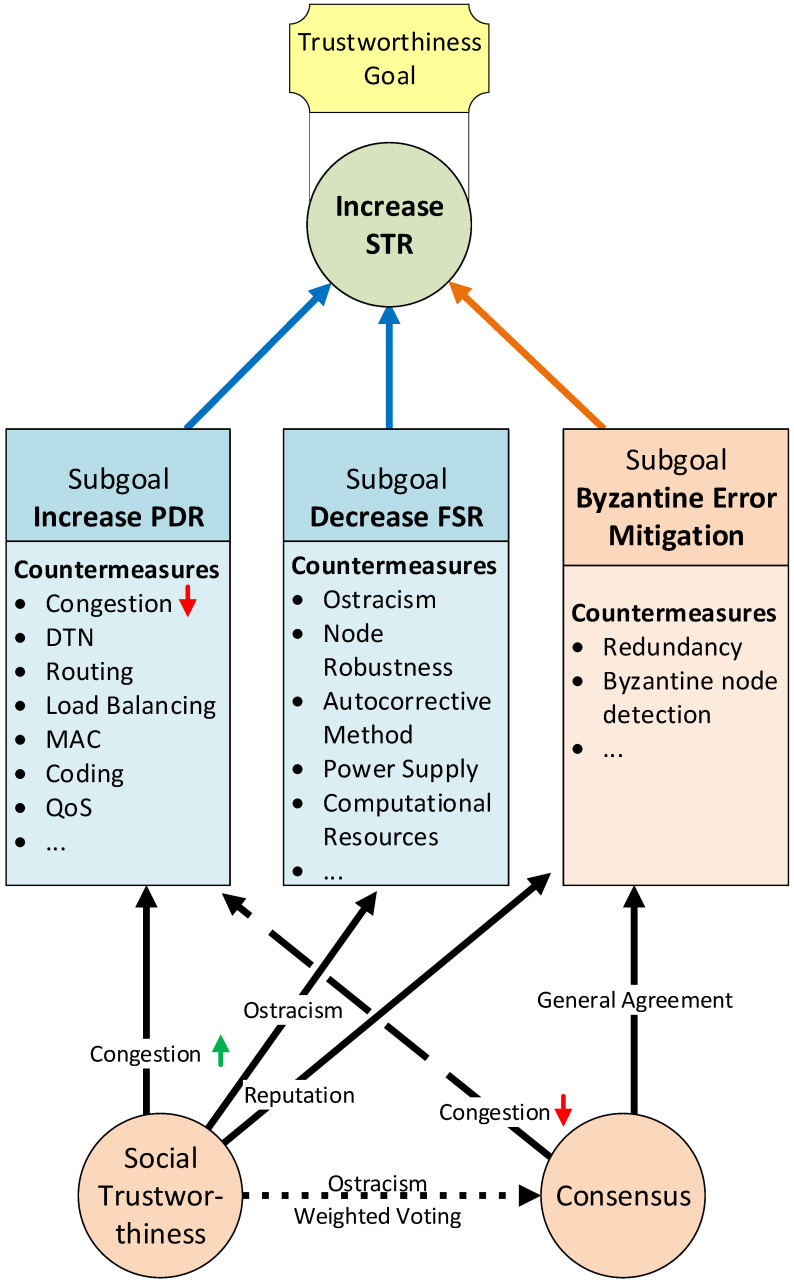
Trustworthiness model goals and countermeasures relationship.

Performed simulations have also led us to understand better how all the proposed model actors work and relate to each other. Figure 8 synthesizes it. Blue-colored elements form part of our model base layers, and orange-colored elements form part of the extension layers. The final goal was to increase the STR to provide better trustworthiness. Three main factors directly help to increase the STR: (1) mitigating/tolerating byzantine errors; (2) decreasing the FSR (2), and (3) increasing the PDR. These factors can be seen as subgoals that leverage the success of the final goal to provide trustworthiness. Each of these subgoals can be accomplished by implementing a set of actions or countermeasures. Each of these countermeasures affects only one of the subgoals. 

Moreover, we have two transversal actions that affect more than one subgoal. These transversal actions implement the extension layers of our model: the Consensus Layer and the Social Trustworthiness Layer. Continuous-line arrows indicate a positive outcome, discontinuous-line arrows indicate a negative outcome, and dotted-line arrows indicate an uncertain outcome. Using social trustworthiness can reduce network congestion thanks to the ostracism of nodes with the worst reputation and send only the values from nodes with the highest reputation to the control center. Social trustworthiness also helps to reduce the FSR thanks to the ostracism of bad reputation nodes. It also leverages the mitigation of byzantine errors because only values from high reputation nodes (leaders) are trusted. 

On the other hand, implementing a consensus mechanism mitigates byzantine errors thanks to the general agreements that are reached by all nodes from a consensus group. Contrarily, the Consensus Layer can negatively affect the PDR, given that it introduces a considerable amount of extra traffic to the network that could lead to link congestion. Finally, the Consensus Layer could also be affected by the Social Trustworthiness Layer if nodes’ reputations were used to increase the reliability of general agreements (e.g., weighted voting based on node’s reputation, ostracism of byzantine nodes), although its exact effect still remains unclear.

Future work aims to study the influence of implementing a DTN architecture at the NVIS backbone network, given that it has characteristics of challenging networks. The authors also plan to study the viability of deploying a FANET in the access network to provide connectivity to sensors placed outside the coverage area of the current LoRa network.

## Figures and Tables

**Table 1:** Qualitative benchmark of the studied trustworthiness approaches.

Trustworthiness Use	[34,35,36]	[38,39,40,41]	[42]	[43]	[44,45]	[47]	[50,51,52,53]	[54,55,56,57,58,59,60]	Own Model
Data Trustworthiness	High	None	None	None	None	None	Medium	Medium	High
Network Trustworthiness	Low	Medium	High	Medium	High	High	Low	Low	High
Social Trustworthiness	None	None	None	None	None	None	High	None	High
Consensus	None	None	None	None	None	None	None	High	High
Metrics used	Faulty Sensed Data	Bit Error Rate	Packet Delivery Ratio (PDR)	PDR, Throughput, Delay	PDR, Delay	PDR, Delay	Successful Transactions	Successful Transaction, Byzantine Node Tolerance, Throughput	Faulty Sensing Ratio, PDR, Successful Transaction Rate, Byzantine Node Tolerance

**Table 2:** Trustworthiness metrics.

Layer	Metric	Range
Data	Faulty Sensing Ratio [35,36]	[0, 1]
Network	Packet Delivery Ratio [44,45]	[0, 1]
Social	Successful Transaction Rate [50,51]	[0, 1]
Consensus	Byzantine Node Tolerance [55,56]	[0, 1]

**Table 3:** Network parameters used to model the scenario.

Parameter	NVIS	LoRa
Transmission Band	4.3 MHz	868 MHz
Channel Bandwidth	2.3 kHz	125 kHz
Channel Bitrate	4.6 kbps	5.47 kbps
Coverage Range	Up to 250 km	Up to 30 km
Daytime Availability (6 a.m.–5 p.m.)	70–100%	100% (LoS), 2–100% (No LoS)
Night Availability (5 p.m.–6 a.m.)	0%	100% (LoS), 2–100% (No LoS)
Maximum Payload Size	242 bytes	140 bytes

**Table 4:** Simulation parameters.

Parameter	Value
Number of runs per test	30
Simulation duration	120 hours (5 days)
Simulation step	1 h
$Pb_{0}$	[1 × 10^−3^, 2 × 10^−3^, 4 × 10^−3^, 8 × 10^−3^, 1 × 10^−2^, 2 × 10^−2^, 4 × 10^−2^, 8 × 10^−2^, 1 × 10^−1^]
*k*	5.7 × 10^−5^
Routing protocol	[AODV, OLSR]
Consensus mechanism	[None, PBFT]
Social Trustworthiness	[True, False]
Number of NVIS gateways	5
Number of GTN-P clusters per gateway	[8, 16, 32, 64, 128, 256, 512, 1024, 2048, 4096]
Number of GTN-P redundant stations per cluster	[1–10]

## Data Availability

Data is contained within the article, it is available based on reasonable request to the authors.

## Appendix A

**Algorithm A1: Sensor Node Application Pseudocode**
**int** t, gateway_id, own_id, pk_id, tx_time, num_retries;
**int** data_values[3 2], node_reputations[N]; //N: number of redundnant nodes
**float** Pb0, Pb, k;
**boolean** consensus, social, is_leader, ack_received;
*initializeVariables*(Pb0,k,consensus,social, gateway_id, own_id, is_leader, ack_received);
**for** (t=0; t++; t<T_MAX){
num_retries = 0;
Pb = Pb0 + k * t;
data_values = *gatherData*(Pb);
**if** (consensus==TRUE){
data_values = *reachGeneralAgreement*(data_values);
**if** (social==TRUE) node_reputations = *computeReputations*();

**if** (*checkLeader*(node_reputations)==TRUE)
[tx_time, pk_id] = *sendPacket*(data_values, gateway_id, own_id);
}**else**{
pk_id = NULL;
}
}**else**{
[tx_time, pk_id] = *sendPacket*(data_values, gateway_id, own_id);
}
**if** (pk_id != NULL){
ack_received = *checkAck*(pk_id);
**while**(ack_received==FALSE && num_retries<MAX_RETRIES){
**if** (*currentTime*() >= tx_time + MAX_TIMEOUT){
[tx_time, pk_id] = *sendPacket*(data_values, gateway_id, own_id);
num_retries++;
}
ack_received = *checkAck*(pk_id);
}
}
pk_id = NULL;
}

**Algorithm A2: Gateway Node Application Pseudocode**
**int** own_id, sensor_id; control_ctr_id, pk_id, tx_time, num_retries;
**int** data_values[32], stored_values[N][32], node_reputations[N]; //N: number of redundnant nodes
**float** Pb;
**boolean** social, ack_received, data_pk_received;
*initializeVariables*(Pb, social, sensor_id, pk_id, own_id, control_ctr_id, ack_received, data_pk_received);
**while**(TRUE){
num_retries = 0;
**if** (*dataPkReceived*()==TRUE){
[sensor_id, pk_id, data_values] = *retrievePkData*();
**if** (social==FALSE){
*sendAck*(pk_id, own_id, sensor_id);
[tx_time, pk_id] = *forwardDataPk*(data_values, gateway_id, sensor_id, control_ctr_id);
ack_received = *checkAck*(pk_id);
**while**(ack_received==FALSE && num_retries<MAX_RETRIES){
**if** (*currentTime*() >= tx_time + MAX_TIMEOUT){
[tx_time, pk_id] = *forwardDataPk*(data_values, gateway_id, sensor_id, control_ctr_id);
num_retries++;
}
ack_received = *checkAck*(pk_id);
}
ack_received=FALSE;
num_retries=0;
}**else**{
stored_values[sensor_id] = data_values;
node_reputations[sensor_id] = *computeReputation*(data_values);
**if** (*roundIsFinished*()==TRUE){
[data_values, sensor_id] = *chooseData*(node_reputations, stored_values);
[tx_time, pk_id] = *forwardDataPk*(data_values, gateway_id, sensor_id, control_ctr_id);
ack_received = *checkAck*(pk_id);
**while**(ack_received==FALSE && num_retries<MAX_RETRIES){
**if** (*currentTime*() >= tx_time + MAX_TIMEOUT){
[tx_time, pk_id] = *forwardDataPk*(data_values, gateway_id, sensor_id, control_ctr_id);
num_retries++;
}
ack_received = *checkAck*(pk_id);
}
ack_received=FALSE;
num_retries=0;
}
}
}
}

**Algorithm A3: Control Center Application Pseudocode**
**int** own_id, sensor_id; gateway_id, pk_id;
**int** data_values[32];
**boolean** data_pk_received;
*initializeVariables*(sensor_id, pk_id, own_id, gateway_id, data_pk_received);
**while**(TRUE){
**if** (*dataPkReceived*()==TRUE){
[sensor_id, gateway_id, pk_id, data_values] = *retrievePkData*();
*storeData*(data_values, sensor_id);
*computeSTR*(data_values);
*sendAck*(pk_id, gateway_id);
}
}
